# Demonstration of a Transparent and Adhesive Sealing Top for Microfluidic Lab-Chip Applications

**DOI:** 10.3390/s24061797

**Published:** 2024-03-11

**Authors:** Anurag Agarwal, Asif Salahuddin, Mohammed Jalal Ahamed

**Affiliations:** MicroNano Mechatronics Laboratory, Department of Mechanical Automotive and Materials Engineering, University of Windsor, Windsor, ON N9B 3P4, Canada; manurag403@gmail.com (A.A.); asifsaldin@gmail.com (A.S.)

**Keywords:** microfluidics, sealing, leak, fluorescence, bonding, cover glass, prototyping

## Abstract

A transparent and adhesive film-based enclosing and sealing method is here presented for out-of-cleanroom-based open-form microfluidic devices. The commercially available polyester flexible film known as Microseal ‘B’ is presented in this paper as a cover seal for open-form microfluidic devices. This film is adaptable to high working temperatures and is biocompatible. The quality of the sealing film was investigated by leak tests, fluorescence tests, and contact angle measurements. The investigations revealed its sealing strength, fluorescence detection compatibility, and surface wettability. It was found that the proposed sealing polyester film on the 3D-printed device could sustain a gauge pressure of 2.7 atm at a flow rate of 4 mL/min without any leaks. It also provided fluorescence detection compatibility and an intensity-to-background ratio in the range of 2.3 to 4.5 for particle sizes of 5 μm and 15 μm, respectively, which is comparable with the performances of other sealing materials. The film’s hydrophobicity is comparable to other polymers used in microfluidics. This paper concludes by showcasing some applications of such transparent tops in classical microfluidic devices used for droplet generation and fluid mixing, in order to demonstrate the prospects of this fabrication technique in lab-on-a-chip devices.

## 1. Introduction

Rapid and outside cleanroom manufacturing processes, such as 3D printing and laser cutting to fabricate microfluidic lab-on-a-chip devices, are catalyzing research and innovation in portable analytical tools, point-of-care diagnostics, and personalized medicine. These processes enable swift design iterations, slashing time and costs in device development. Classical microfabrication techniques, such as lithography [1,2,3,4,5,6], can provide precision fabrication of lab-on-a-chip (LOC) devices; however, they often involve a longer turnaround time and higher cost. Rapid and out-of-cleanroom-based manufacturing of fluidic lab-on-a-chip (LOC) devices is increasingly gaining attention due to the compatibility of this approach with fabrication flowcharts in commonplace industrial facilities. Hence, it can improve on the otherwise poor accessibility and scalability of microfluidic cleanroom prototyping attempts. Various out-of-cleanroom-based fabrication techniques, such as laser writing [7], micromachining [8], injection molding [9,10] and hot embossing [11], are pursued because of their lower cost and faster turnaround time. In addition, some of these manufacturing methods also allow multilayer device fabrication. The fabrication of multilayer devices using classical microfabrication techniques is complex, and often not possible. Multilayer devices produced with layer-by-layer photolithography need secured bonding between multiple layers. The adhesive bonding method or conventional thermal compression bonding is challenging and usually requires ultrasonic [12], edge laser wielding [13], or chemical treatment [14] to enhance the bonding procedure.

As an alternative to the fabrication of bonding-based multilayer devices, direct fabrication of microfluidic devices with 3D printing [15] is becoming popular because of its ability to print multiple layers simultaneously [16]. Along with the fabrication methods, the material used for the fabrication is also important. In early lab-on-a-chip devices, glass was the main material for fabrication [17,18], but emphasis shifted toward elastomers and thermal plastics, generally designated polymers [19]. Elastomers such as polydimethylsiloxane (PDMS) [20] and thermoplastic polymers such as polycarbonate [20] or polymethyl methacrylate (PMMA) [21,22] are low-cost. For example, PDMS is attractive for its biocompatibility, low toxicity, chemical inertness, and mechanical flexibility [23,24], etc. The 3D printing method is taking incremental steps to enable bulk manufacturing of LOC devices with such polymer materials. It offers enhanced volumetric efficiency, high chip density, and quick reconfigurability via computer-aided design (CAD). Different types of 3D printing processes relevant to microfluidic applications are reviewed in [25,26,27,28], and the pros and cons of these processes are discussed in those papers. Each printing-based manufacturing system differs from others and the applications of 3D-printed microfluidic devices are versatile in biochemical, tissue printing, fluidic dispensing, mixing, and electrochemical detectors [29,30,31,32,33,34,35,36,37,38,39,40,41,42,43]. Additionally, as an alternative method to gas chromatography and mass spectroscopy systems, 3D-printed, straight, and serpentine channels have been fabricated with PDMS and plasma bonded with the cover layer [36] to analyze gas sensitivity and selectivity.

In many of these 3D-printed devices, optical clarity is an important index for ranking microfluidic devices, as it enables on-chip classification and quantification of chemical and biological reactions. Optical access in general is important for many fluidic and biochemical experimentations for studying fluid behavior or molecular interactions by fluorescence because it allows for real-time monitoring, rapid quantification of lamina mixing, and reaction kinetics. Three-dimensional printing utilizing some resins can provide transparency [44,45], but those methods still struggle to yield higher throughput and are on the expensive side of this technology. Therefore, researchers are developing innovative solutions using different types of transparent films, such as glass, PDMS, or adhesive tapes [46,47], to seal open microchannels for optical access. Various adhesive transparent films have been used for sealing microfluidic devices [48,49,50,51], laser-cut devices [52], microchannels on biaxially oriented polystyrene (BOPS) [53], and multilayer microfluidic devices [54]. In some cases, both PDMS film and adhesive film are tested to seal different sections [55] of the 3D-printed device. However, although these transparent sealing alternatives (for example, cover glass or PDMS) provide better optical access but need complex bonding or sealing mechanisms, finding simple transparent sealing mechanisms suitable for 3D-printed or laser-cut open channel microfluidics remains a challenge.

The majority of 3D-printed devices discussed above are non-transparent or opaque, which renders them unsuitable for fluidic applications requiring optical access. Open-form 3D-printed devices, when sealed with a transparent top, can offer optical access, yet the leak-free sealing of the top remains an issue. Pressure-driven continuous microfluidics necessitate device integrity to prevent leaks. These challenges limit the applicability of 3D-printed microfluidics. Therefore, the community is actively seeking solutions that allow for easy sealing while maintaining excellent optical access from the top, all while ensuring device integrity and biocompatibility. In response to this gap, we propose a biocompatible material capable of not only sealing open-form microfluidic devices but also providing optical access. In this paper, the significant contributions to addressing the research gaps emanated from testing the suitability of a transparent, polyester adhesive flexible film for enclosing 3D-printed devices while providing optical access and substantiating its potential. The proposed commercially available adhesive film was previously used for sealing PCR well plates [54] during the amplification of DNA. But to our knowledge, it has not been used with 3D-printed fluidic lab-on-a-chip devices yet. This polyester adhesive film is highly biocompatible, shows good optical clarity on the PCR well plates, and has a wide working temperature range. It is free of biomass, easy to handle, and not expensive [54]. It has been used for thermal amplification of DNA [55]. The film’s adhesive layer is pressure-sensitive adhesive (PSA). We compared the effectiveness of this adhesive film against some other sealing materials used on our proposed 3D-printed device and observed satisfactory results. The leak test, fluorescence test, and contact angle measurement test discussed in this paper corroborate these claims. We also delved into the applicability of this sealing technique on devices such as a droplet generator and a mixer and will discuss the findings. As a secondary approach, a laser-cutting method was engaged in one of the microfluidic devices, which was also compatible with our method as proposed in this paper.

In Section 2, we provide the details of the materials and methods and provide the required references to the hardware and software for interested readers to find the specifications from the manufacturer names and references. In Section 3, we focus more on the methodology side as we go further into the discussion of the fabrication procedures. This ensures that the workflow is well understood and will be reproducible by other research groups. In Section 4, we analyze different performance characteristics of this film specimen; that is, its adequacy for use as a transparent top layer of a 3D printed microfluidic device. In Section 5, we showcase the applications of such transparent tops on some microfluidic devices, e.g., a device used for droplet generation and a fluid mixing device. Finally, in Section 6, conclusions are drawn from the observations, and some perspectives are laid out, which should guide research in the future.

## 2. Materials and Methods

Our microfluidic devices were manufactured using a low-cost desktop-based 3D printer named ‘Ultimaker 3 Extended’ (Utrecht, Netherlands). It uses a dual extrusion method and is capable of water-soluble support with polyvinyl alcohol (PVA) material if necessary. The layer resolution was up to 20 μm. The extrusion speed was 16 mm^3^/s, and the travel speed was 30–300 mm/s. The XY positioning precision was 12.5 μm, and the z positioning precision was 2.5 μm. The software package SolidWorks 2020 was used to create the computer-aided design (CAD) solid model for the microfluidic device, and afterward, the model was loaded on the printer. The 3D-printed device used low toxicity, highly biodegradable polylactic acid (PLA) as the printing material. In addition to 3D-printed microfluidic devices, we also tested the adhesive film with laser cut microfluidic devices. The laser-cut device used acrylic glass, which has a high melting point and low cost.

The Microseal ‘B’ Model #MSB1001 (Bio-Rad Laboratories Inc., Hercules, CA, USA) adhesive sealswere used for sealing the targeted devices in fabrication. The film has a thickness of 200 μm. The film, being transparent, is used in high-sensitivity optical assays. The working temperature of the film ranges from −40 °C to 110 °C. It is free of DNA, RNA, and other biomass, which enables its usage in biological experiments. The proposed adhesive layer was compared with conventional cover-glass-based sealing. The cover glass ‘Model #170-C2260’ used for the transparency test was sourced from Ultident Scientific, Inc (Saint-Laurent, QC, Canada). It had dimensions of 22 mm × 60 mm and had a thickness of 140 μm. A low-thickness cover glass was chosen to minimize the effect of diffraction in the experimental measurement. A Dino-Lite Digital Microscope ‘Model #AM4115T-GFBW’ was used for taking images/videos. To pump the fluid into the chip, a syringe pump ‘Model #Pump11 Elite’ was used and was sourced from Harvard Apparatus, Holliston, MA, USA.

For consistency in demonstrating results, in this paper, the Microseal ‘B’ from Bio-Rad Laboratory (Hercules, CA, USA) is referred to as ‘Bio-Rad’ film, and the cover glass ‘Model #170-C2260’ is referred to as ‘cover-glass’ in subsequent sections in this paper.

## 3. Fabrication Procedure

**Figure 1:**
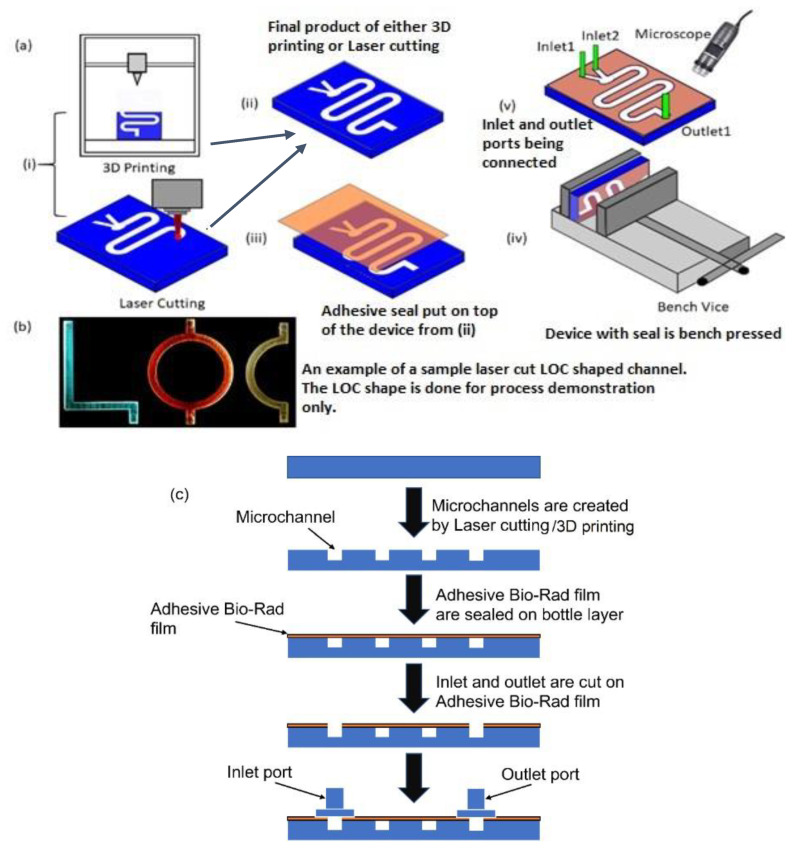
(**a**) Schematic of step-by-step fabrication of microfluidic device with adhesive film on the top to seal the device: (i) 3D printing and laser cutting can be used to obtain the required chip (ii); the final output of both the manufacturing process; (iii) the adhesive film is used to seal the top of the device; (iv) the whole device is pressed using a bench-vice; (v) the inlet and outlet connectors are connected to the device and the whole setup is observed under a microscope. (**b**) A picture of LOC-shaped channel made by laser cutting and flowing colored dye. (**c**) Simple schematic flowchart of the step-by-step fabrication process.

To fabricate the open-form microfluidic device layer, additive manufacturing techniques, such as 3D printing, and subtractive manufacturing techniques, such as laser cutting (Figure 1a (i)), were used. Figure 1a (ii) shows the fabricated channel with dimensions of 60 mm (L) × 5 mm (W) with a height of 5 mm, which was manufactured either with 3D printing or with laser cutting. Before placing the transparent seal on the top of the fabricated device, holes were made in the seal for inlet and outlet port positions. Next, the device was sealed with the proposed adhesive film (Figure 1a (iii)) so that optical transparency was achieved, and no fluid was able to leak out from the channel during the experiment. The transparent adhesive film was instrumental in providing optical access to the chip through the top.

Three different ways of attaching the adhesive Bio-Rad film were investigated. The first method was carried out by pressing the film on top of the device to seal it in place. In the second method, the film was simply placed on the top for self-seal without using any pressing mechanism, and in the third method, the attachment was achieved with the additional aid of a pressed double-sided tape. This tape had carrier layers coated on both sides with adhesives. Loose attachment of the film to the device layer could result in air gaps, which will make the bond weak. To avoid such a problem, the complete device was pressed in a bench vice for approximately 10 to 15 min (Figure 1a (iv)). Ensuring a higher seal strength also meant that the inlet and outlet connectors could be connected to the channel more securely. After the top of the device was sealed, inlet and outlet ports were glued to the device. Fluidic tubing connected the inlet with the syringe pump. The tubing used for connecting the syringe with the device had inner and outer diameters of ~0.8 mm and ~2.4 mm, respectively. The assembly process was observed under a microscope (Figure 1a (v)) to locate any possible voids. Figure 1b shows a laser-cut chip sealed with the proposed transparent film, and different-colored dyes were pumped into letter-shaped channels of varying color. Figure 1c depicts a simple flow chart of the fabrication process. It is to be noted that creating holes in the seal before attaching it to the fabricated device vs. creating the holes on the seal after the seal was placed on the device did not make a difference to the robustness of the sealing process.

## 4. Results and Discussion

To investigate the usability of this Bio-Rad film on any microfluidic device, it was essential to characterize the effectiveness of this film as a transparent top layer of a 3D-printed microfluidic device. The performance of this film was compared to the performances of other widely used transparent top-layer materials. The tests we performed were the leak test, fluorescence test, and wettability test, outlined in the next subsections.

### 4.1. Leak Test

The fluidic channel (Figure 1a) in the test had the dimensions of 60 mm (L) × 5 mm (W) with a height of 5 mm. During the leak test, the syringe pump was run to operate the syringe to push the fluid at a flow rate of 4 mL/min through the inlet valve. The outlet valve was connected to the pressure gauge via a pressure valve. The valve ensured that the outlet was blocked and no liquid could escape. For each type of sealing method, the experiments were repeated 4 or 5 times, with uncertainties that were acceptable within the pressure gauge’s sensitivity guidelines. The bench-top vice handle was given equal turns to press the Bio-Rad film onto the device for each of the repeatability tests, to ensure an equal amount of pressure was applied on each occasion.

**Figure 2:**
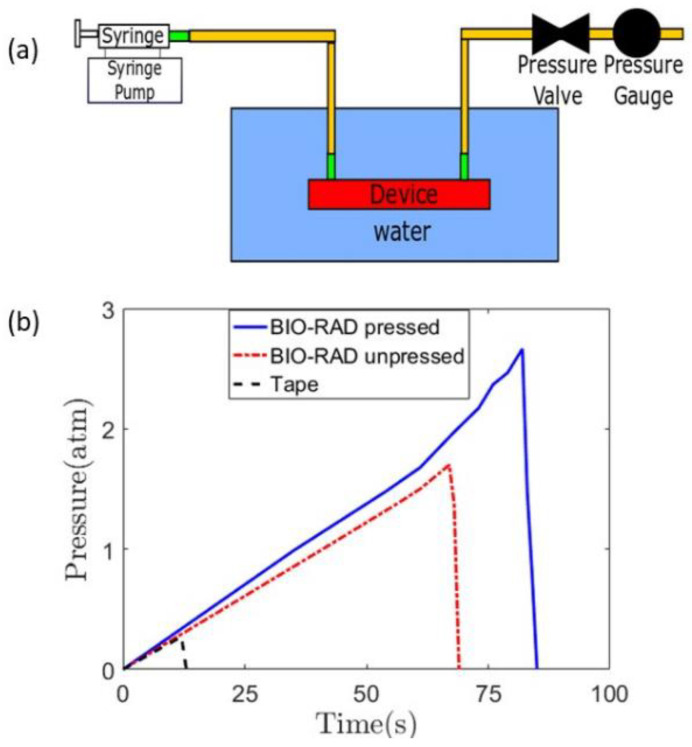
(**a**) Complete setup of a leak test, with a syringe pump infusing the working fluid while a pressure gauge is attached to measure the pressure at the outlet of the channel. The microfluidic device is placed inside a water beaker. (**b**) Quantification of the leak test for three different cases, that is, Bio-Rad pressed, unpressed, and with scotch double-sided tape.

To record the time of the initiation of a leak, the entire device was placed inside a water beaker, as shown in Figure 2a. The initiation of the leak produced rising bubbles toward the top that could be easily observed. The pressure gauge reading showed an abrupt drop as the leak released pressure (Figure 2b).

The leak test studies were performed on three cases: Bio-Rad pressed, Bio-Rad unpressed, and using double-sided tape with Bio-Rad. When the Bio-Rad film was not pressed with a vice, the device leaked at a lower pressure compared to when it was pressed using a vice. In all cases, the pressure increased linearly from zero-gauge pressure to a higher pressure in a non-leaking device. The leaks occurred at gauge pressures of 2.7 atm, 1.7 atm, and 0.27 atm for Bio-Rad pressed, Bio-Rad unpressed, and double-sided tape with Bio-Rad, respectively. The bench-pressed method for sealing the Bio-Rad film showed the highest potential to be used in a high-pressure microfluidic application, exhibiting the least susceptibility to leakage. Therefore, it can be concluded that applying pressure during the assembly of the device yields better leak protection. The unpressed Bio-Rad leak test showed lower capability than the pressed one, but it did show higher leak protection capability than that of the double-sided tape-based device.

Additionally, since in microfluidics the flow is laminar in nature, we assume that the pressure drop inside the channel will be proportional to the flow rate when the channel dimensions and fluid properties remain the same. This will enable the user of the device to investigate the performance of the strength of the seals linearly as well without undertaking a set of testing experiments.

### 4.2. Fluorescence Microscopy Application

The technique of fluorescence microscopy is an essential tool in microfluidic devices applied in biology and biomedical sciences, as well as in biochemical materials science. It renders attributes that are not readily available in other contrast mode imaging with traditional optical microscopy. The application of an array of fluorochromes can make it possible to identify cells, molecules, and submicroscopic cellular components with a high degree of specificity amid fluorescing materials. Fluorescence imaging plays an important role in microfluidic-based labs-on-a-chip. Therefore, it is essential to validate the fluorescence imaging applicability of a 3D printed device using the adhesive seal when there will be a microfluidic sample inside this device, and fluorescence microscopy would be the preferred method for optical investigation. The digital microscope used in this research can image particles involving fluorescent objects with blue LEDs for excitation at 480 nm. It has a 510 nm emission filter that is designed to observe green fluorescence.

**Figure 3:**
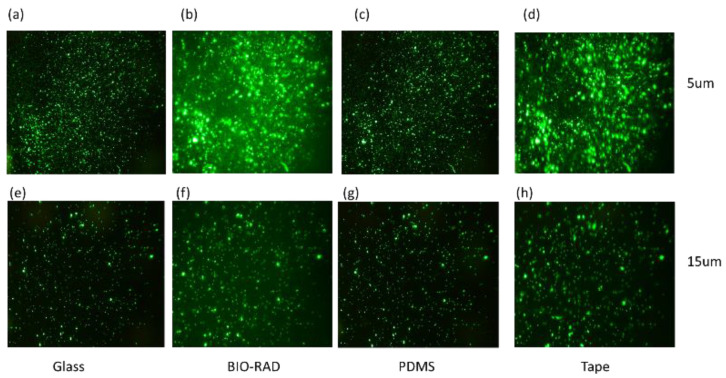
Particles with sizes of 5 μm (**a**–**d**) and 15 μm (**e**–**h**) are observed under a microscope covering the sample through materials such as glass, Bio-Rad, PDMS, and tape.

To study the quality of fluorescence imaging through the top seal cover, we separately loaded the microfluidic channel with fluorescence particle samples with a particle size of either 5 μm or 15 μm. The samples were observed under a microscope through four different types of top covers, namely, thin cover glass, Bio-Rad, PDMS and double-sided tape. Figure 3a–d shows the images of the 5 μm particles, and in Figure 3e–h, the images of the 15 μm particles are shown, which correspond to the setup for devices with different top seals.

Figure 3 shows fluorescence images of 5 μm particles (Figure 3a–d) and 15 μm particles (Figure 3e–h) imaged through various transparent top sealing materials. While investigating the fluorescence images of Figure 3, images corresponding to 5 μm particles may seem brighter than those of the 15 μm particles because of the higher concentration of 5 μm particles in the field of view, and this may lead to the conclusion that Figure 3a–d might have higher optical clarity than Figure 3e–h. The images are not filtered with a common background base. Therefore, to find the exact signal intensities, a quantitative analysis was needed to study the intensity-to-background ratio, giving scope to some additional observations in Table 1.

In Table 1, the IBR values are calculated using a commercial software ‘ImageJ (version 1.51)’. The intensity is calculated as the variance of pixel values of the total image, and the background is calculated by selecting a particular region (loc, scale). We can make a few observations, such as: (i) IBR for 15 µm > 5 µm; (ii) IBRs for glass and PDMS are higher, and those for Bio-Rad and double-sided tape are lower. This competitive result for the Bio-Rad IBR compared to those of other widely used microfluidic seals confirms that Bio-Rad is an acceptable choice as a top seal material for fluorescence imaging. It indicates that as the particle size increases, the optical performance of the device with Bio-Rad top seal improves.

Additionally, the optical performance of a sealing material may also depend on the variations in its thickness, and this aspect could be of relevant research interest as well. The working distance (W.D.) of high-magnification microscopy is generally small. Depending on the magnification of the objective lens, it could be less than 0.3 mm. The contradiction between the thickness of microfluidic chips and the recommendation for the working distance of high-resolution microscopy prevents microfluidic devices from being further exploited in many applications [56]. Since, in our test, the range of thickness for the four materials being tested was considerably low (140 µm to 200 µm), we expected that the observation of the fluorescence particles in our device with a thin observation interface via the object of high numerical aperture would be sufficient for any optical interrogation. The thickness of the film could be selected based on the target optical application.

### 4.3. Wettability Test

In microfluidic applications, along with optical properties, the fluidic wettability of the working surface or walls is often important for fluidic characterizations. The contact angle measurement is a standard way to understand the wettability phenomenon. The contact angle is the angle, conventionally measured through the liquid, where a liquid–vapor interface meets a solid surface.

This measurement quantifies the wettability of a solid surface by a liquid. The contact angle plays an important role in understanding the behavior of a liquid on a material in many microfluidic applications, for example, droplet generation in a T-junction [57], fluid transport on a microfluidic platform [58], and displacement of immiscible droplets in a channel [59,60]. Therefore, studying the contact angle for the proposed sealing layer is important for its potential application in different microfluidic platforms.

**Figure 4:**
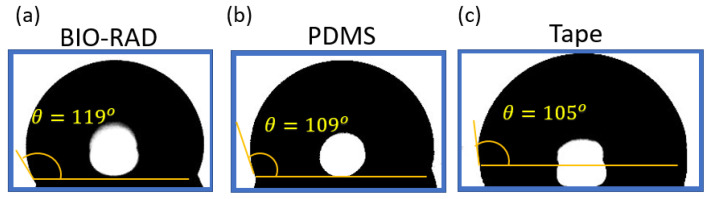
Contact angle *θ* of DI water on (**a**) Bio-Rad, (**b**) PDMS, and (**c**) tape, showing comparable hydrophobicity.

Figure 4 shows the contact angle measurement of DI (di-ionized) water on four different materials. If the water contact angle is smaller than 90° (measured through water, that is the liquid side), the solid surface is considered hydrophilic; and if the water contact angle is larger than 90°, the solid surface is considered hydrophobic. To make the measurement, a droplet of DI water is placed on each of the concerned materials using a pipette. Images are taken with a microscope with the backgrounds brightly illuminated using a light source from the opposite end. After taking pictures, the images are processed to find the liquid interface as well as to calculate the corresponding contact angle with the tangent method.

The uncertainty of using a tangent method to estimate the contact angle was analyzed in [61]. Using a standard error propagation technique involving partial derivatives, it was proven that the contact angle measurements for sessile drops are prone to a small error of ≤±2°. For contact angles (*θ*) < 60°. However, as *θ* values approached 90°, uncertainty increases asymptotically and can exceed ±5°. From Figure 4, it is apparent that our measurements were within the range of 100° < *θ* < 120°; so conservatively, we can say the uncertainty in measurements was ±5°.

Figure 4 shows that the contact angle of water on the Bio-Rad material was the highest (119°) compared to the contact angle of water on other materials, such as glass, PDMS, and tape. This shows that Bio-Rad is a hydrophobic material like many other polymers. Microfluidic devices, such as droplet generators, require the contact surfaces to be hydrophobic. Therefore, Bio-Rad is a compatible material to be used in a 3D-printed droplet generator microfluidic device.

In the next section, we apply the proposed transparent sealing layer in some classical microfluidic applications to showcase its device-level performance.

### 4.4. Device Reuse Test Scope

A great number of microdevices are suitable for single use only, and it is difficult to recover the contents inside the microchannels or perform advanced microscopy visualization due to their irreversible sealing method. On the flip side, conventional reversible sealing procedures are only suitable for low pressures (less than 35 kPa), and chips are prone to debonding or leaking [62]. Irreversible sealing methodologies are useful due to their strong bonding and ability to support high pressures. For example, with our irreversible sealing process of attaching the top seal with the 3D microfluidic device, we could achieve 2.7 atm gauge pressure. However, the reusability and reversibility of the film can be studied further.

## 5. Applications

In this section, we will discuss some applications of our method to demonstrate the viability of the new sealing top in creating applied microfluidic devices. We selected several classical microfluidic devices, including droplet generators and fluid mixers, that are applied widely in many research and development applications. All the devices were placed parallel to the ground and sealed with Bio-Rad pressed adhesive films.

T-junction-based droplet generators are used in many microfluidic applications [63], such as in vitro compartmentalization [64], the production of polymeric particles [65], and the development and screening of biological assays [66]. Thus, they are among the classical and widely used types of devices that are important to study with our method.

**Figure 5:**
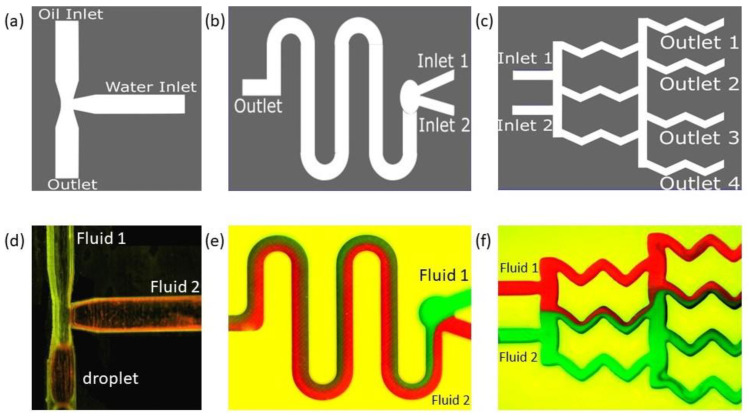
Schematic and results of our 3D-printed classical microfluidic devices sealed with the transparent adhesive Bio-Rad film to create (**a**) a droplet generator, (**b**) a diffusion mixer (serpentine shape), and (**c**) a diffusion mixer (gradient mixer). Device designs are shown in the top row, and the experimental fluid flows through corresponding designs are shown in the bottom row (**d**–**f**). Note: experiments were performed when the devices were parallel to the ground.

A conventional T-junction-based droplet generator design is shown in Figure 5a. In our droplet generator, silicone oil (Fluid 1) was used as the continuous phase fluid, and distilled water (Fluid 2) was used as the dispersed phase fluid in a T-channel. The reason for considering DI water as a dispersed fluid and silicone oil (mineral oil) as a continuous phase was due to their immiscibility, which aids in breaking up the dispersed fluid near the T-junction as a droplet. The reason for using DI water as a working fluid was that most of the time, droplet microfluidics are used in electrical sensing applications. If we are using electrical sensing, such as a capacitor-based sensor to sense droplets that happen to be charged, the possible ions in the fluid could produce unwanted peaks and noise in the measurement. Hence, DI water was used, because it aids in measuring only the pure material properties between dispersed and continuous fluid. The properties of the fluids are provided in Appendix A.

Oil and water were introduced from two different inlets (Figure 5a) orthogonal to each other. The water-dispersed phase with a flow rate of 4mL/min was sheared by the continuous oil phase with a flow rate of 8 mL/min at the junction, and water droplets were generated. In a T-junction such as this, droplets are formed in three different regimes [67]: jetting, dripping, and squeezing. We observed droplets in the squeezing regime (Figure 5d). In the squeezing regime, the stream of the dispersed phase obstructed the main channel and caused a spike in the upstream pressure. It squeezes the neck of the emerging droplets and initiates breakup to reach a stable state where droplet coalescence will be unlikely. In our experiment, the droplets formed were squeezed and not spherical; hence, finding the eccentricity of the shape became important. Using image analysis, the eccentricity value was found to be 0.88, which was close to an ellipse.

In the next application, we prepared microfluidic devices used for mixing fluids. In the context of microfluidics, the mixing process is identified as a phenomenon that brings uniformity in concentration. Conventional bench-top mixing procedures used at macroscopic levels, such as stirring the fluid to create turbulence, are impracticable for small-scale LOC systems. This is due to limitations and complexities in micro-actuation techniques attaining larger values of Reynolds numbers (critically ≥2000 to 2300) in fluid systems with very small dimensions. A small Reynolds number (<10) means an extremely weak inertial force that is unable to cause any rapid turbulent mixing.

Many micromixing techniques have been developed and are primarily categorized as ‘active’ or ‘passive’ methods. Passive micromixers utilize manipulation of the pressure head using various passive geometric features as the only source of energy input to drive the fluid at a constant rate. The strategy is to utilize the laminar characteristics of the flow to induce lamination and to generate chaotic advection, which in turn increases the contact surface and contact time between the species flows. In our device, the embedded barriers/obstructions along the flow create flow disruptions to introduce weak inertia and extend the entire effective width of the channel.

**Figure 6:**
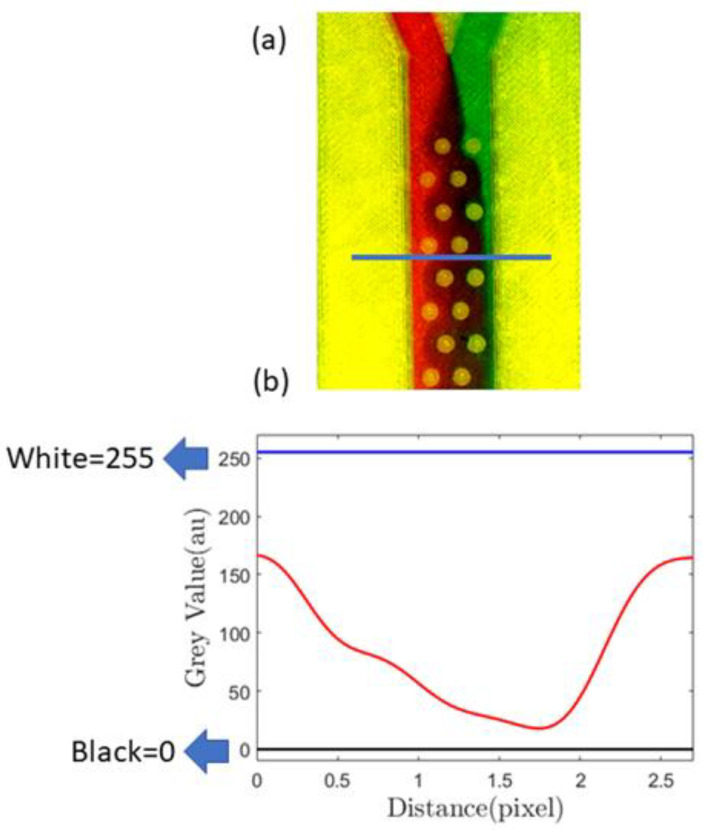
(**a**) Image showing mixing using passive barriers inside the channel, the blue line showing the cross-section where mixing characterization was performed; (**b**) quantification of the mixing using a grayscale value calculated at the position of the blue line [drawn in (**a**)], blue lines on (**b**) showing black and white greyscale values.

In Figure 6a, we demonstrate this passive mixing device that uses the proposed sealing film. The passive mixer uses cylindrical barriers to enhance mixing. A similar device was studied in detail using both the passive and active methods in our previous paper [68]. In this paper, however, we show various experimental results obtained with passive mixing methodology only. To measure the mixing efficiency, we first identified a cross-sectional position sufficiently away from the inlet, and a horizontal line designates that position (Figure 6a). In Figure 6b, the minima and maxima of the grayscale value corresponding to black and white are 0 and 255, respectively, and the red line indicates mixing.

**Figure 7:**
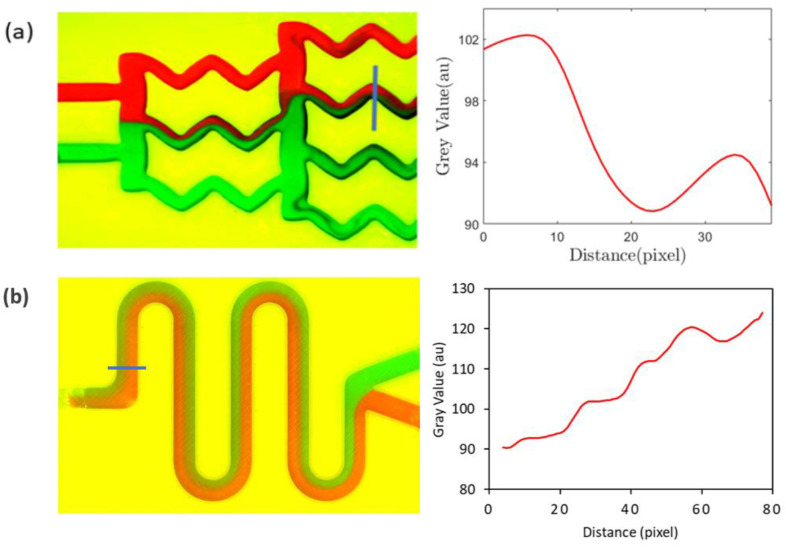
Quantification of the mixing for (**a**) the gradient mixer and (**b**) the serpentine mixer using the grayscale values obtained on the blue lines drawn across a channel leg for the mixer. Red and green color represents two different fluids.

After mixing initiates, red and green streams of fluids combine to give a brown hue, for which the grayscale value is lower than 255. Therefore, when there is more mixing, the grayscale value decreases, and the area under the red line in Figure 6b decreases. The results from a similar type of analysis are demonstrated in Figure 7, the geometries of which correspond to the gradient mixer (Figure 5b) and the serpentine mixer (Figure 5c).

In contrast to passive mixers, active mixing devices improve the mixing performance by using external force on the fluid flow to accelerate the diffusion process and by inducing stronger turbulence than a passive mixer can offer. Often mechanical or electrical actuation mechanisms are microfabricated inside the device to impose active mixing. In general, various actuation mechanisms have been developed, for example, moving actuators, acoustic/ultrasonic, dielectrophoretic, magnetic, thermal electrohydrodynamic force-based actuators, etc. Electroosmotic active mixing is one of the widely investigated mixing techniques, because of its ease of implementation and faster mixing performance. In our numerical simulation paper [68] on electroosmotic mixing, flow actuation is achieved by applying an electric voltage that is controlled by specially placed electrodes inside a channel. The proposed sealing technique was investigated with passive mixers, but is expected to behave similarly for active mixing, such as in an electroosmotic flow-driven active mixer, as far as device integrity, sealing, and optical clarity are concerned, because the Bio-Rad is not electrochemically active.

## 6. Conclusions

In this work, we demonstrated a new transparent sealing top with the possibility of application in microfluidic devices fabricated by additive-manufacturing 3D printing or laser cutting tools with an easy-to-assemble process. The film, being transparent, helped the experimenters to perform optical inspections. The film is also biocompatible, low-cost, and easy to handle. Several tests, such as the leak test, the fluorescence test, and the wettability test, were carried out on microfluidic devices fabricated using the sealing top to compare the capabilities of the film against other cover materials. Our results showed that the film can sustain a gauge pressure as high as 2.7 atm, implying its effectiveness for usage in high-pressure microfluidic applications. The film is hydrophobic in nature and is suitable for use in droplet generators based on that requirement. The intensity-to-background ratio analysis of the fluorescence microscopy images showed the proposed film is comparable to PDMS and cover glass for use in applications requiring optical access. Some microfluidic applications of the devices, made with the 3D-printing method and with the selected sealing methods, are showcased herein, demonstrating the potential for such a suite in this field. Further research opportunities include assessing the effect of film thickness on the optical performance of these devices, and innovating ways to reuse (reverse) the 3D-printed devices with efficient removal of the transparent tops so as not to damage the devices. This technology has strong application potential in the fields of microfluidic devices for biological, medical, and analytical chemistry, due to its simple and flexible design process, reduced lead time, and low-cost high-end offerings in both research and industrial setup.

## Figures and Tables

**Table 1:** The raw intensity-to-background (IBR) ratio. A higher value indicates higher intensity.

Film Material	Thickness (µm)	IBR (5 µm Particles)	IBR (15 µm Particles)
Cover-glass	140	6.69	9.45
PDMS	~200	6.17	9.12
Bio-Rad	200	2.27	4.47
Double-sided Tape	~140	4.22	6.66

## Data Availability

The data that support the findings of this study are available upon reasonable request from the authors.

## Appendix A. Fluid Properties

Nominal values at room temperature and pressure conditions:

DI water: density = 1000 kg/m^3^

Dynamic viscosity = 0.001 Pa·S

Mineral oil: density = 894.5 kg/m^3^

dynamic viscosity = 0.02085 Pa·S

Interfacial tension between DI water and mineral oil: 40 mN/m

## References

[1] Duffy D.C., McDonald J.C., Schueller O.J.A., Whitesides G.M. (1998). Rapid Prototyping of Microfluidic Systems in Poly(dimethylsiloxane). Anal. Chem..

[2] Xia Y., Whitesides G. (1998). Soft Lithography. Annu. Rev. Mater. Sci..

[3] Chou S.Y., Krauss P.R., Renstrom P.J. (1996). Nanoimprint lithography. J. Vac. Sci. Technol. B Microelectron. Nanometer Struct. Process. Meas. Phenom..

[4] Deckman H.W., Dunsmuir J.H. (1982). Natural lithography. Appl. Phys. Lett..

[5] Switkes M., Rothschild M. (2001). Immersion lithography at 157 nm. J. Vac. Sci. Technol. B Microelectron. Nanometer Struct. Process. Meas. Phenom..

[6] Chen C., Hirdes D., Folch A. (2003). Gray-scale photolithography using microfluidic photomasks. Proc. Natl. Acad. Sci. USA.

[7] Liao Y., Cheng Y., Liu C., Song J., He F., Shen Y., Chen D., Xu Z., Fan Z., Wei X. (2013). Direct laser writing of sub-50 nm nanofluidic channels buried in glass for three-dimensional micro-nanofluidic integration. Lab Chip.

[8] Bossink E.G.B.M., Vollertsen A.R., Loessberg-Zahl J.T., Van der Meer A.D., Segerink L.I., Odijk M. (2022). Systematic characterization of cleanroom-free fabricated macrovalves, demonstrating pumps and mixers for automated fluid handling tuned for organ-on-chip applications. Microsyst. Nanoeng..

[9] McCormick R., Nelson R., Alonso-Amigo M., Benvegnu D., Hooper H. (1997). Microchannel Electrophoretic Separations of DNA in Injection-Molded Plastic Substrates | Analytical Chemistry. Anal. Chem..

[10] Gale B.K., Jafek A.R., Lambert C.J., Goenner B.L., Moghimifam H., Nze U.C., Kamarapu S.K. (2018). A Review of Current Methods in Microfluidic Device Fabrication and Future Commercialization Prospects. Inventions.

[11] Lin T.-Y., Do T., Kwon P., Lillehoj P.B. (2017). 3D printed metal molds for hot embossing plastic microfluidic devices. Lab Chip.

[12] Zhang Z., Luo Y., Wang X., Zheng Y., Zhang Y., Wang L. (2009). Thermal assisted ultrasonic bonding of multilayer polymer microfluidic devices. J. Micromech. Microeng..

[13] Li J., Liu C., Ke X., Xu Z., Li M., Duan Y., Fan Y., Wang L. (2012). Fabrication of a thermoplastic multilayer microfluidic chip. J. Mater. Process. Technol..

[14] Zhang H., Liu X., Li T., Han X. (2014). Miscible Organic Solvents Soak Bonding Method Use in a PMMA Multilayer Microfluidic Device. Micromachines.

[15] Bhattacharjee N., Urrios A., Kang S., Folch A. (2016). The upcoming 3D-printing revolution in microfluidics. Lab Chip.

[16] Waldbaur A., Rapp H., Lange K., Rapp B. (2011). Let there be chip—Towards rapid prototyping of microfluidic devices: One-step manufacturing processes. Anal. Methods.

[17] Harrison D.J., Fluri K., Seiler K., Fan Z., Effenhauser C.S., Manz A. (1993). Micromachining a Miniaturized Capillary Electrophoresis-Based Chemical Analysis System on a Chip. Science.

[18] Harrison D.J., Manz A., Fan Z., Luedi H., Widmer H.M. (1992). Capillary electrophoresis and sample injection systems integrated on a planar glass chip. Anal. Chem..

[19] Becker H., Locascio L.E. (2002). Polymer microfluidic devices. Talanta.

[20] Chang C.W., Cheng Y.J., Tu M., Chen Y.H., Peng C.C., Liao W.H., Tung Y.C. (2014). A polydimethylsiloxane–polycarbonate hybrid microfluidic device capable of generating perpendicular chemical and oxygen gradients for cell culture studies. Lab Chip.

[21] Toossi A., Moghadas H., Daneshmand M., Sameoto D. (2015). Bonding PMMA microfluidics using commercial microwave ovens. J. Micromech. Microeng..

[22] Sia S.K., Whitesides G.M. (2003). Microfluidic devices fabricated in Poly(dimethylsiloxane) for biological studies. Electrophoresis.

[23] McDonald J., Whitesides G. (2002). Poly(dimethylsiloxane) as a Material for Fabricating Microfluidic Devices. Acc. Chem. Res..

[24] Mata A., Fleischman A.J., Roy S. (2005). Characterization of Polydimethylsiloxane (PDMS) Properties for Biomedical Micro/Nanosystems. Biomed. Microdevices.

[25] Nielsen A.V., Beauchamp M.J., Nordin G.P., Woolley A.T. (2020). 3D Printed Microfluidics. Annu. Rev. Anal. Chem..

[26] He Y., Wu Y., Fu J.Z., Gao Q., Qiu J.J. (2016). Developments of 3D Printing Microfluidics and Applications in Chemistry and Biology: A Review. Electroanalysis.

[27] Gross B.C., Erkal J.L., Lockwood S.Y., Chen C., Spence D.M. (2014). Spence Evaluation of 3D Printing and Its Potential Impact on Biotechnology and the Chemical Sciences. Anal. Chem..

[28] Kitiara G., Dimitri P. (2023). 3D printed microfluidics for bioanalysis: A review of recent advancements and applications. TrAC Trends Anal. Chem..

[29] Femmer T., Kuehne A.J., Wessling M. (2014). Print your own membrane: Direct rapid prototyping of polydimethylsiloxane. Lab Chip.

[30] Costantini M., Jaroszewicz J., Kozoń Ł., Szlązak K., Święszkowski W., Garstecki P., Guzowski J. (2019). 3D-Printing of Functionally Graded Porous Materials Using On-Demand Reconfigurable Microfluidics. Angew. Chem. Int. Ed..

[31] Goh W.H., Hashimoto M. (2018). Dual Sacrificial Molding: Fabricating 3D Microchannels with Overhang and Helical Features. Micromachines.

[32] Hardin J.O., Ober T.J., Valentine A.D., Lewis J.A. (2015). Microfluidic Printheads for Multimaterial 3D Printing of Viscoelastic Inks. Adv. Mater..

[33] Tumbleston J.R., Shirvanyants D., Ermoshkin N., Janusziewicz R., Johnson A.R., Kelly D., Chen K., Pinschmidt R., Rolland J.P., Ermoshkin A. (2015). Continuous liquid interface production of 3D objects. Science.

[34] Au A.K., Bhattacharjee N., Horowitz L.F., Chang T.C., Folch A. (2015). 3D-printed microfluidic automation. Lab Chip.

[35] Rogers C.I., Qaderi K., Woolley A.T., Nordin G.P. (2015). 3D printed microfluidic devices with integrated valves. Biomicrofluidics.

[36] Aghaseyedi M., Salehi A., Valijam S., Shooshtari M. (2022). Gas Selectivity Enhancement Using Serpentine Microchannel Shaped with Optimum Dimensions in Microfluidic-Based Gas Sensor. Micromachines.

[37] Kolesky D.B., Truby R.L., Gladman A.S., Busbee T.A., Homan K.A., Lewis J.A. (2014). 3D Bioprinting of Vascularized, Heterogeneous Cell-Laden Tissue Constructs. Adv. Mater..

[38] Pavan Kalyan B.G., Kumar L. (2022). 3D Printing: Applications in Tissue Engineering, Medical Devices, and Drug Delivery. AAPS Pharm. Sci. Tech..

[39] Zhang Q., Bei H.P., Zhao M., Dong Z., Zhao X. (2022). Shedding light on 3D printing: Printing photo-crosslinkable constructs for tissue engineering. Biomaterials.

[40] Symes M.D., Kitson P.J., Yan J., Richmond C.J., Cooper G.J., Bowman R.W., Cronin L. (2012). Integrated 3D-printed reactionware for chemical synthesis and analysis. Nat. Chem..

[41] Kitson P.J., Rosnes M.H., Sans V., Dragone V., Cronin L. (2012). Configurable 3D-Printed millifluidic and microfluidic ‘lab on a chip’ reactionware devices. Lab Chip.

[42] Quintanilla A., Vega G., López P., García F., Madurga E., Belmonte M., Casas J.A. (2021). Enhanced Fluid Dynamics in 3D Monolithic Reactors to Improve the Chemical Performance: Experimental and Numerical Investigation. Ind. Eng. Chem. Res..

[43] Erkal J.L., Selimovic A., Gross B.C., Lockwood S.Y., Walton E.L., McNamara S., Spence D.M. (2014). 3D printed microfluidic devices with integrated versatile and reusable electrodes. Lab Chip.

[44] Tian K., Bae J., Bakarich S.E., Yang C., Gately R.D., Spinks G.M., Vlassak J.J. (2017). 3D Printing of Transparent and Conductive Heterogeneous Hydrogel–Elastomer Systems. Adv. Mater..

[45] Urrios A., Parra-Cabrera C., Bhattacharjee N., Gonzalez-Suarez A.M., Rigat-Brugarolas L.G., Nallapatti U., Samitier J., DeForest C.A., Posas F., Garcia-Cordero J.L. (2016). 3D-printing of transparent bio-microfluidic devices in PEG-DA. Lab Chip.

[46] Bonyár A., Sántha H., Ring B., Varga M., Kovács J.G., Harsányi G. (2010). 3D Rapid Prototyping Technology (RPT) as a powerful tool in microfluidic development. Procedia Eng..

[47] Comina G., Suska A., Filippini D. (2014). Low cost lab-on-a-chip prototyping with a consumer grade 3D printer. Lab Chip.

[48] Fan Y., Liu S., Gao K., Zhang Y. (2018). Fully enclosed paper-based microfluidic devices using bio-compatible adhesive seals. Microsyst. Technol..

[49] Martinez A.W., Phillips S.T., Carrilho E., Thomas S.W., Sindi H., Whitesides G.M. (2008). Simple Telemedicine for Developing Regions: Camera Phones and Paper-Based Microfluidic Devices for Real-Time, Off-Site Diagnosis. Anal. Chem..

[50] Yu J., Wang S., Ge L., Ge S. (2011). A novel chemiluminescence paper microfluidic biosensor based on enzymatic reaction for uric acid determination. Biosens. Bioelectron..

[51] Yu P., Deng M., Yang Y. (2019). New Single-Layered Paper-Based Microfluidic Devices for the Analysis of Nitrite and Glucose Built via Deposition of Adhesive Tape. Sensors.

[52] Ren Y., Ray S., Liu Y. (2019). Reconfigurable Acrylic-tape Hybrid Microfluidics. Sci. Rep..

[53] Fan Y., Wang H., Liu S., Liu J., Gao K., Zhang Y. (2018). Rapid prototyping of shrinkable BOPS-based microfluidic devices. Microfluid. Nanofluid..

[54] Fan Y., Liu S., He J., Gao K., Zhang Y. (2018). Rapid prototyping of flexible multilayer microfluidic devices using polyester sealing film. Microsyst. Technol..

[55] Boos J.A., Beuvink I. (2016). Whole-Body Scanning PCR, a Tool for the Visualization of the In Vivo Biodistribution Pattern of Endogenous and Exogenous Oligonucleotides in Rodents.

[56] Chen X., Hou S., Chu J., Xiong Y., Xiong P., Liu G., Tian Y. (2017). Observation Interface of PDMS Membrane in a Microfluidic Chip Based on One-Step Molding. Micromachines.

[57] Boruah M., Sarker A., Randive P., Pati S., Chakraborty S. (2018). Wettability-mediated dynamics of two-phase flow in microfluidic T-junction. Phys. Fluids.

[58] Ghosh A., Ganguly R., Schutzius T., Megaridis C. (2014). Wettability patterning for high-rate, pumpless fluid transport on open, non-planar microfluidic platforms—Lab on a Chip (RSC Publishing). Lab Chip.

[59] Kang Q., Zhang D., Chen S. (2002). Displacement of a two-dimensional immiscible droplet in a channel. Phys. Fluids.

[60] Kang Q., Zhang D., Chen S. (2005). Displacement of a three-dimensional immiscible droplet in a duct. J. Fluid Mech..

[61] Extrand C.W. (2016). Uncertainty in contact angle measurements from the tangent method. J. Adhes. Sci. Technol..

[62] Camilo P.-S., Ana B.P.-P., Gustavo R., Natalia B., Aparna A., Shekhar B., Maximiliano S.P., Betiana L. (2022). Novel Reproducible Manufacturing and Reversible Sealing Method for Microfluidic Devices. Micromechanics.

[63] Seemann R., Brinkmann M., Pfohl T., Herminghaus S. (2011). Droplet based microfluidics. Rep. Prog. Phys..

[64] Zhu Y., Power B.E., Werther M., Seitz H. (2008). Lab-on-a-Chip In Vitro Compartmentalization Technologies for Protein Studies Protein—Protein Interaction Advances in Biochemical Engineering/Biotechnology.

[65] Dendukuri D., Doyle P.S. (2009). The Synthesis and Assembly of Polymeric Microparticles Using Microfluidics. Adv. Mater..

[66] Theberge A., Courtois F., Schaerli Y., Fischlechner M., Abell C., Hollfelder F., Huck W. (2010). Microdroplets in Microfluidics: An Evolving Platform for Discoveries in Chemistry and Biology. Angew. Chem. Int. Ed..

[67] Donvito L., Galluccio L., Lombardo A., Morabito G., Nicolosi A., Reno M. (2015). Experimental validation of a simple, low-cost, T-junction droplet generator fabricated through 3D printing. J. Micromech. Microeng..

[68] Agarwal A., Salahuddin A., Wang H., Ahamed M.J. (2020). Design and development of an efficient fluid mixing for 3D printed lab-on-a-chip. Microsyst. Technol..

